# Transcriptome Profiling of Wild *Arachis* from Water-Limited Environments Uncovers Drought Tolerance Candidate Genes

**DOI:** 10.1007/s11105-015-0882-x

**Published:** 2015-04-11

**Authors:** Ana C. M. Brasileiro, Carolina V. Morgante, Ana C. G. Araujo, Soraya C. M. Leal-Bertioli, Amanda K. Silva, Andressa C. Q. Martins, Christina C. Vinson, Candice M. R. Santos, Orzenil Bonfim, Roberto C. Togawa, Mario A. P. Saraiva, David J. Bertioli, Patricia M. Guimaraes

**Affiliations:** Embrapa Recursos Genéticos e Biotecnologia, Parque Estação Biológica, 02372 Final W5 Norte, Brasília, DF Brazil; Embrapa Semiárido, Petrolina, PE Brazil; CONAB, Brasília, DF Brazil; Universidade de Brasília, Campus I, Brasília, DF Brazil

**Keywords:** SSH libraries, 454 Sequencing, Differential gene expression, Dry-down, Peanut wild relatives, qRT-PCR

## Abstract

Peanut (*Arachis hypogaea* L.) is an important legume cultivated mostly in drought-prone areas where its productivity can be limited by water scarcity. The development of more drought-tolerant varieties is, therefore, a priority for peanut breeding programs worldwide. In contrast to cultivated peanut, wild relatives have a broader genetic diversity and constitute a rich source of resistance/tolerance alleles to biotic and abiotic stresses. The present study takes advantage of this diversity to identify drought-responsive genes by analyzing the expression profile of two wild species, *Arachis duranensis* and *Arachis magna* (AA and BB genomes, respectively), in response to progressive water deficit in soil. Data analysis from leaves and roots of *A. duranensis* (454 sequencing) and *A. magna* (suppression subtractive hybridization (SSH)) stressed and control complementary DNA (cDNA) libraries revealed several differentially expressed genes in silico, and 44 of them were selected for further validation by quantitative RT-PCR (qRT-PCR). This allowed the identification of drought-responsive candidate genes, such as Expansin, Nitrilase, *NAC*, and *bZIP* transcription factors, displaying significant levels of differential expression during stress imposition in both species. This is the first report on identification of differentially expressed genes under drought stress and recovery in wild *Arachis* species. The generated transcriptome data, besides being a valuable resource for gene discovery, will allow the characterization of new alleles and development of molecular markers associated with drought responses in peanut. These together constitute important tools for the peanut breeding program and also contribute to a better comprehension of gene modulation in response to water deficit and rehydration.

**Electronic supplementary material:**

The online version of this article (doi:10.1007/s11105-015-0882-x) contains supplementary material, which is available to authorized users.

## Introduction

Peanut (*Arachis hypogaea* L.) is an oilseed crop cultivated worldwide and one of the major grain legumes in tropical and subtropical regions. However, its productivity is strongly affected in drought-prone areas by water scarcity. Drought is one of the most serious constraints to crop production and, associated with the predicted consequences of global climate change, increases the need for drought-adapted varieties. Considering the highly complex aspects of drought tolerance in plants, the understanding of response mechanisms to water-limited conditions and identification of the pathways and genes involved in these responses are strategic for the development of new varieties (Hu and Xiong 2014; Juenger 2013).

In this respect, transcriptome approaches have the potential to aid the understanding of tolerance mechanisms and to orientate gene discovery. Moreover, high-throughput sequencing allows the study of simultaneous gene expression and their regulation profiles in different biological processes, thus enabling species comparisons and revealing key information regarding evolution divergence and domestication processes (Li et al. 2012; Madlung and Wendel 2013). Therefore, large transcriptome surveys have been undertaken in recent years for many crop plants, including peanut (Chen et al. 2013; Wu et al. 2013; Yin et al. 2013; Zhang et al. 2012).

Peanut has a large tetraploid genome, and its genetic studies have been hindered over the years by the scarcity of genomic resources (Brasileiro et al. 2014; Holbrook et al. 2011; Pandey et al. 2012) such as physical maps, a comprehensive transcriptome database, and a fully sequenced genome, which is now on the verge of being accomplished by the International Peanut Genome Initiative (IPGI; http://www.peanutbioscience.com/homepage.html). In addition, only recently, studies identifying genes associated with drought stress responses using transcriptome approaches for the genus *Arachis* have become available (Ding et al. 2014; Govind et al. 2009; Guimaraes et al. 2012; Jain et al. 2001; Li et al. 2014; Luo et al. 2005; Ranganayakulu et al. 2011).

Unlike the cultivated species, wild *Arachis* is more genetically diverse having undergone selection through evolution to adapt to a wide variety of habitats and conditions and is more likely to harbor resistance/tolerance to biotic and abiotic stresses (Bertioli et al. 2011). In particular, the genus *Arachis* is endemic to South America being mostly associated with tropical savannah regions, characterized by a marked dry season during the southern winter. In particular, *Arachis duranensis* and *Arachis magna*, diploid wild annual species with AA and BB *Arachis* genomes, respectively, are native to low rainfall regions in Bolivia and Argentina (Krapovickas and Gregory 2007; Leal-Bertioli et al. 2012; Robledo et al. 2009). Indeed, previous studies showed that the accession K7988 of *A. duranensis* and KG30097 of *A. magna* displayed the most conservative transpiration profile under water-limited conditions when compared to other genotypes including wild, cultivated, and synthetic amphidiploids *Arachis* (Leal-Bertioli et al. 2012), making them very interesting genotypes for drought-related gene discovery. Moreover, these two species are also the parentals of the mapping populations used to develop the diploid A and B genetic maps (Moretzsohn et al. 2005, 2009), which are fundamental for the localization of candidate genes in genetic reference maps and co-localization with QTLs (Leal-Bertioli et al. 2009).

In addition, the availability of a comprehensive *A. duranensis* root transcriptome under drought stress database developed by our group (Guimaraes et al. 2012) directed the selection of *A. duranensis* together with *A. magna*, as representatives of the AA and BB genomes of *Arachis* and good candidates to identify drought-responsive genes in the present study*.* The analysis of deep-sequencing-based transcriptome profiling of both genotypes submitted to gradual soil water deficit enabled the in silico identification of many differentially expressed genes, from which, 44 candidates were selected for further quantitative RT-PCR (qRT-PCR) validation. The data herein constitutes a novel genomic resource for wild *Arachis* available for the development of new molecular markers and gene discovery, thus facilitating the transfer of important traits into cultivated peanut varieties.

## Materials and Methods

### Soil Gradual Water Deficit (Dry-Down) Experiment

Seeds of *A. duranensis* (accession K7988) and *A. magna* (accession KG30097) were obtained from the Embrapa Active Germplasm Bank (Brazil) and germinated in germitex paper with 2 % (*w*/*v*) Ethrel (2-chloroethylphosphonic acid) and 0.05 % (*w*/*v*) thiram (tetramethylthiuram disulfide). Plantlets were transferred to pots (one per pot) containing 500 g of soil and kept regularly watered in a greenhouse.

Dry-down experiments were carried out using 3-month-old plants, as previously described (Leal-Bertioli et al. 2012). Briefly, plants were divided into two sets: one submitted to dry-down (drought-stressed (DS) plants), and another, the control group, was regularly watered (well-watered (WW) plants). To prevent water evaporation from soil, the pots were bagged, leaving the plant stem and leaves outside the plastic bag. Individual transpiration rate (TR) was estimated gravimetrically, every day, and normalized transpiration rate (NTR) calculated as the ratio between TR values and the average TR for the first 3 days for each individual plant, as described by Muchow and Sinclair (1991). Greenhouse temperature and relative air humidity were recorded hourly using an Electronic Datalogger Sato SK-L200TH II (Sato, Japan).

Leaves and roots of *A. duranensis* and *A. magna* were collected at different points, based on the average NTR of DS plants, corresponding to different stages of water deficit and re-watering: (i) point 1, beginning of drought stress (NTR values between 0.65–0.60); (ii) point 2, representing the rapid decline in the TR of DS plants (NTR values around 0.4); (iii) point 3, considered the endpoint for the water deficit treatment (NTR values below 0.3); (iv) point 4, plant re-watering to 70 % field capacity (FC) followed by sample collection after 30 min for *A. duranensis* and 5 h for *A. magna*; and (v) point 5, only *A. duranensis* plants collected 72 h after re-watering, when DS plants showed a phenotype similar to the WW plants. For both species, samples from leaves and roots of 15 DS plants, and another 15 from WW, were harvested at each collecting point, immediately frozen in liquid nitrogen and stored at −80 °C.

### RNA Extraction

Extraction of total RNA was conducted using a modified lithium chloride protocol, as previously described (Morgante et al. 2011). Total RNA was further purified on Invisorb Plant RNA Mini columns (InViTek, Berlin, Germany), according to the manufacturer’s instructions. RNA quality/integrity was checked on ethidium-bromide-stained 1.5 % (*w*/*v*) agarose gels and quantified using a NanoDrop® ND-1000 spectrophotometer (Thermo Scientific, Waltham, USA). RNA samples were subsequently treated with DNase (TURBO DNA-free™, Ambion, USA), according to the manufacturer’s instructions. Total RNA extracted from *A. duranensis* plants was used exclusively for sample preparation for qRT-PCR analysis whereas RNA from *A. magna* plants was used for both complementary DNA (cDNA) libraries construction and qRT-PCR analysis.

### cDNA Library Construction and Sequence Analysis

Sequences from *A. duranensis* cDNA libraries previously obtained by our group (Guimaraes et al. 2012) were used for differential expression analysis and drought-responsive gene mining. The number of raw sequences mapping from each library (WW and DS) to the resulting clusters of de novo transcript assembly (Guimaraes et al. 2012) was used as an estimate of transcript quantity using the “gene counts” model, as previously described (Beneventi et al. 2013). Genes that showed distinct numbers of counts between the two libraries and similarity to drought-stress-responsive sequences in BLASTX algorithm (Altschul et al. 1990) were selected for qRT-PCR analysis.

*A. magna* subtractive cDNA libraries were constructed using WW and DS leaf RNA samples, assembled at equal amounts from collecting points 1, 2, and 3. PolyA+ RNA was subsequently isolated from 3 mg of each RNA assemblage using Oligotex® kit (Qiagen, Hilden, Germany). Suppression subtractive hybridization (SSH) libraries were produced using PCR-Select™ cDNA Subtraction Kit (Clontech, Mountain View, USA) in both directions; i.e., cDNA from stressed plants was used as the driver [given rise to stressed (STR) library] and, afterward, as the tester [control (CTR) library]. Subtractive PCR products were sequenced by the Sanger method using an ABI Prism 3700 DNA Analyzer (Applied Biosystems Inc., Foster City, USA). *A. magna* expressed sequence tags (ESTs) had their quality checked before being trimmed and clustered following Telles and Silva (2001). Functional annotation of *A. magna* ESTs for known proteins/genes was conducted as for *A. duranensis* (Guimaraes et al. 2012), using the BLASTX algorithm (Altschul et al. 1990) followed by Blast2GO tool for functional category assignment (Gotz et al. 2008).

### Gene Validation by qRT-PCR

A first series of differential expression analysis between DS and WW plants by qRT-PCR used *A. duranensis* and *A. magna* samples gathered in pools. For this, leaf and root RNA samples extracted from five individuals at collecting points 1, 2, and 3 were pooled at equal amounts, constituting a biological replicate with 15 samples. For each treatment (DS and WW) and species, three independent replicates were formed. RNA was quality checked and treated with DNase as described above and used for first-strand cDNA synthesis according to Morgante et al. (2011).

qRT-PCR was performed in three technical replicates using the Platinum® SYBR® Green qPCR SuperMix-UDGw/ROX kit (Invitrogen, Carlsbad, USA), on the ABI 7300 Real-Time PCR System (Applied Biosystems, Foster City, USA). Reactions and primer design were conducted as previously described (Morgante et al. 2013). Efficiencies for each primer pair and optimal cycle threshold (Cq) values were estimated using the online real-time PCR Miner tool (Zhao and Fernald 2005). Average Cq values were normalized to two reference genes, for each species, as previously established (Morgante et al. 2011). Expression ratios of messenger RNA (mRNA) transcripts in DS samples relative to WW were determined and statistically tested using REST 2009 v. 1 software (Pfaffl et al. 2002).

Eight genes were selected as candidates for more detailed expression study using a further series of qRT-PCR analysis. For this, samples from each collecting point and tissue from five individuals were pooled in equal amounts to constitute a biological replicate. Three independent replicates with 15 samples each were thus formed per collecting point (1 to 5), treatment (DS and WW), and tissue (leaf and root). The assembled RNA was treated as described above.

## Results and Discussion

### Dry-Down Experiments

**Fig. 1:**
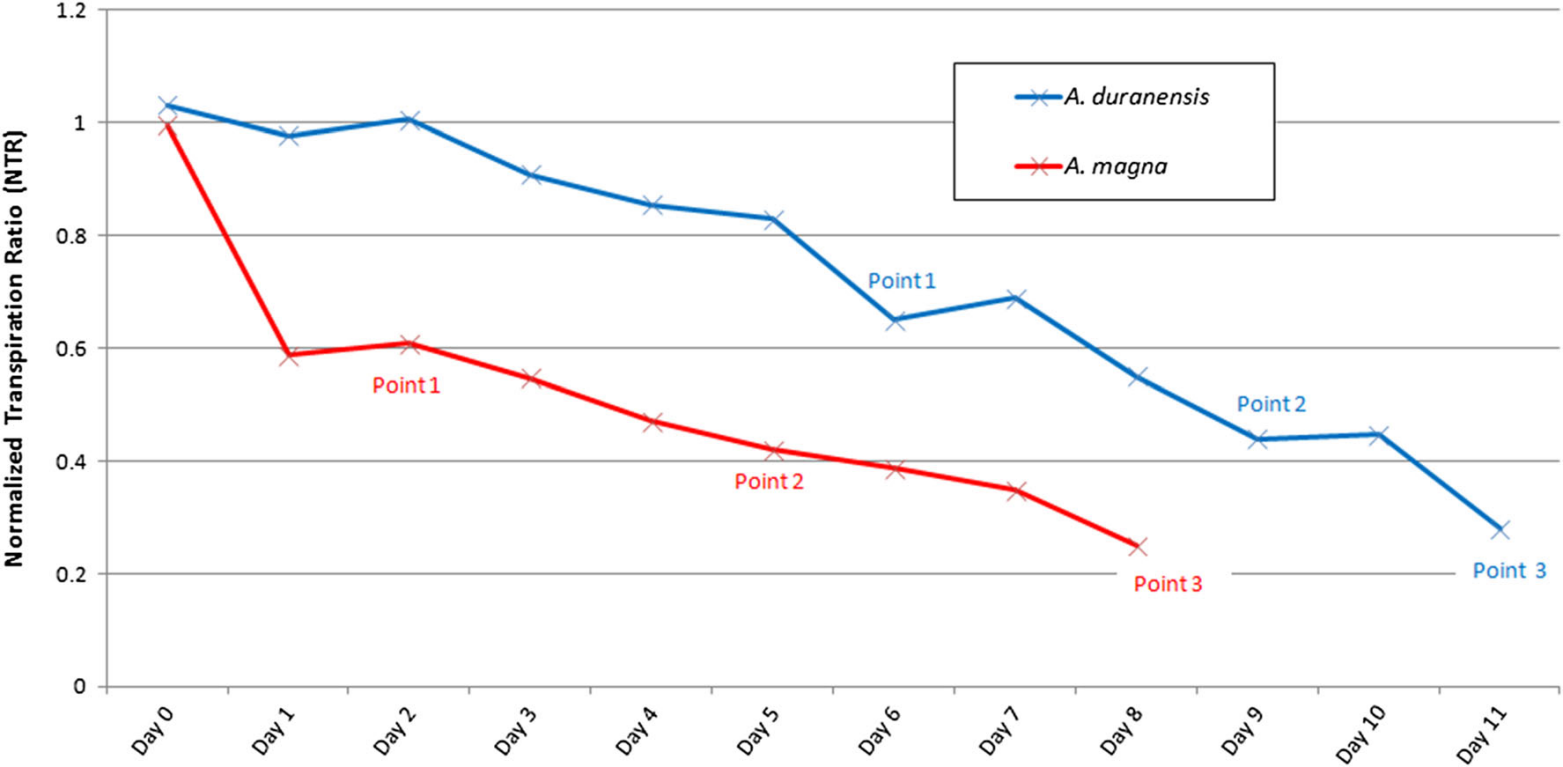
Normalized transpiration ratio (NTR) of *A. duranensis* and *A. magna* during dry-down and after soil re-watering. Day 0 corresponds to dry-down start. Collecting points (1 to 3) are showed in *blue* for *A. duranensis* and in *red* for *A. magna*

The transpiration profile of *A. duranensis* and *A. magna* during gradual water deficit experiments is represented by the NTR behavior in Fig. 1. NTR curves corresponding to overall transpiration data obtained by gravimetrical measurements followed, for both wild species, the typical pattern previously described for *Arachis* and other tropical legume species (Leal-Bertioli et al. 2012; Sinclair and Ludlow 1986; Zaman-Allah et al. 2011). NTR values for both species were adjusted to values close to 1 (Fig. 1) in order to have a similar transpiration behavior for the beginning of the dry-down. With the water deficit imposition, NTRs for DS plants gradually decreased (Fig. 1), reflecting the changes in transpiration patterns, as lower water availability in soil induces the reduction of plant transpiration rates (Sinclair and Ludlow 1986). Therefore, to better capture gene expression modulations associated with the variations in transpiration profile, samples from both species were collected at three time points (collecting points 1 to 3; Fig. 1) in an attempt to provide representative stages of limited water stress, as described by Sinclair and Ludlow (1986). Subsequent collecting points (4 and 5) corresponded to DS plant drought recovery stages after soil rehydration to 70 % FC. During the experiments, temperature ranged from 22 to 31 °C and relative air humidity from 70 to 80 %.

**Fig. 2:**
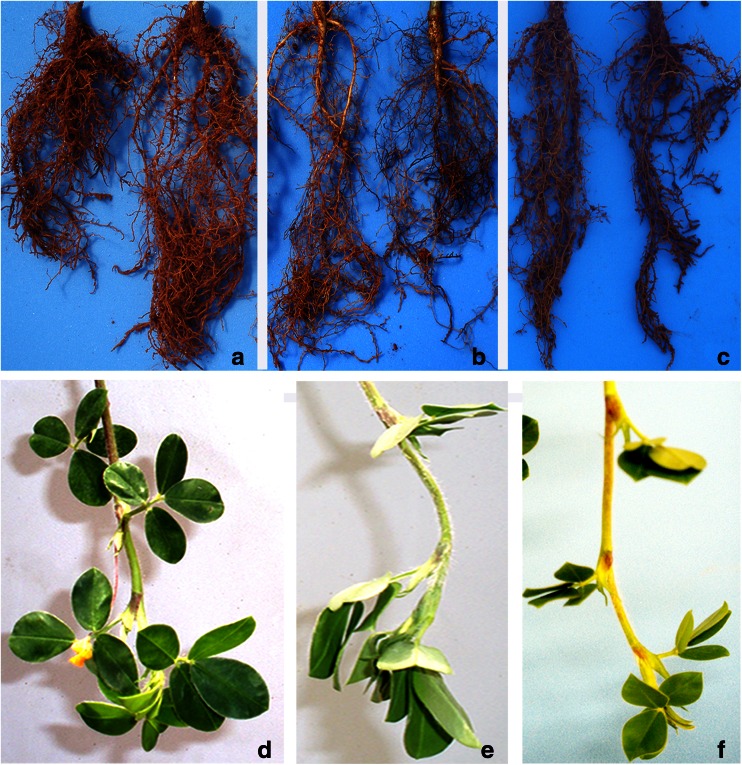
**a**–**f** Roots of *A. duranensis* from two WW plants (**a**) and from two DS plants (**b**) collected at point 3 (NTR 0.28) and from two DS plants (**c**) collected at point 5 (72 h after soil re-watering). Each root corresponds to an independent individual. Leaves of *A. magna* from one WW plant (**d**) and from one DS plant (**e**) collected at point 3 (NTR 0.25) and from one DS plant (**f**) collected at point 4 (5 h after soil re-watering). Each aerial part corresponds to an independent individual. For both species, DS roots (**b**) and leaves (**e**) show a phenotype strongly affected by drought imposition when compared to normal WW plants (**a**, **d**), such as a dehydrated appearance, roots with evident desiccation, brownish and brittle appearance (**b**), and wilting leaflets with chlorotic areas (**e**). The phenotype of re-watered roots (**c**) and leaves (**f**) is partially recovered (WW alike) after 72 and 5 h, respectively

*A. duranensis* drought imposition lasted 11 days. During the stress, transpiration rates of DS plants gradually declined and samples were collected at NTR values of 0.65, 0.44, and 0.28 (Fig. 1). At NTR 0.28 (point 3), *A. duranensis* DS plants displayed a dehydrated appearance, with wilting leaflets with chlorotic areas and roots with evident desiccation, appearing brownish and brittle whereas WW plants displayed a normal phenotype (Fig. 2a, b). Therefore, point 3 was selected as the point in which the drought condition threshold was reached. Following this point, soil of DS plants was fully rehydrated and samples were collected after 30 min (point 4) and 72 h (point 5) of re-watering. At point 5, DS plants recovered transpiration rates close to WW (NTR = 0.92), with leaf and root phenotypes resembling those of controls (Fig. 2c).

*A. magna* drought imposition lasted 8 days and NTR values of DS plants dropped sharply at the first day of dry-down and then declined slightly every day during the subsequent days (Fig. 1). *A. magna* DS samples were collected at the NTR 0.61, 0.42, and 0.25 (points 1 to 3, respectively; Fig. 1). At NTR 0.25, established as the threshold point for the imposed stress, only mild symptoms of dehydration in leaves and roots were observed in DS plants compared to WW (Fig. 2d, e). Remarkably, *A. magna* DS plants recovered the phenotype similar to the WW just 5 h after re-watering (Fig. 2f).

The different time necessary for each species to reach similar NTR under the gradual water deficit herein imposed (Fig. 1) suggests that specific mechanisms of drought responses or physiological differences could be inherent to each genotype. In fact, this assumption is corroborated by previous results showing that *A. duranensis* and *A. magna* transpiration patterns under drought stress conditions differ from each other, yet with the more “conservative” behavior for water use than other *Arachis* genotypes (Leal-Bertioli et al. 2012). *A. duranensis* and *A. magna* grow in low rainfall areas (Krapovickas and Gregory 2007; Leal-Bertioli et al. 2012), and their conservative transpiration performance under limited water availability might be associated with phenotypic plasticity for adaptation to such condition, typical of their native environment. Along with other physiological mechanisms, linked to adaptive phenotypic plasticity, the variation in transpiration rates potentially allows plant endurance during drought stress conditions (Juenger 2013; Zaman-Allah et al. 2011). Therefore, dry-down experiments enabled a detailed comparative analysis of *A. duranensis* and *A. magna* transpiration behavior and also an accurate root and leaf sampling during gradual soil water deficit to be used in comprehensive transcriptome analysis.

### *A. duranensis* Sequence Analysis and Functional Annotation

**Fig. 3:**
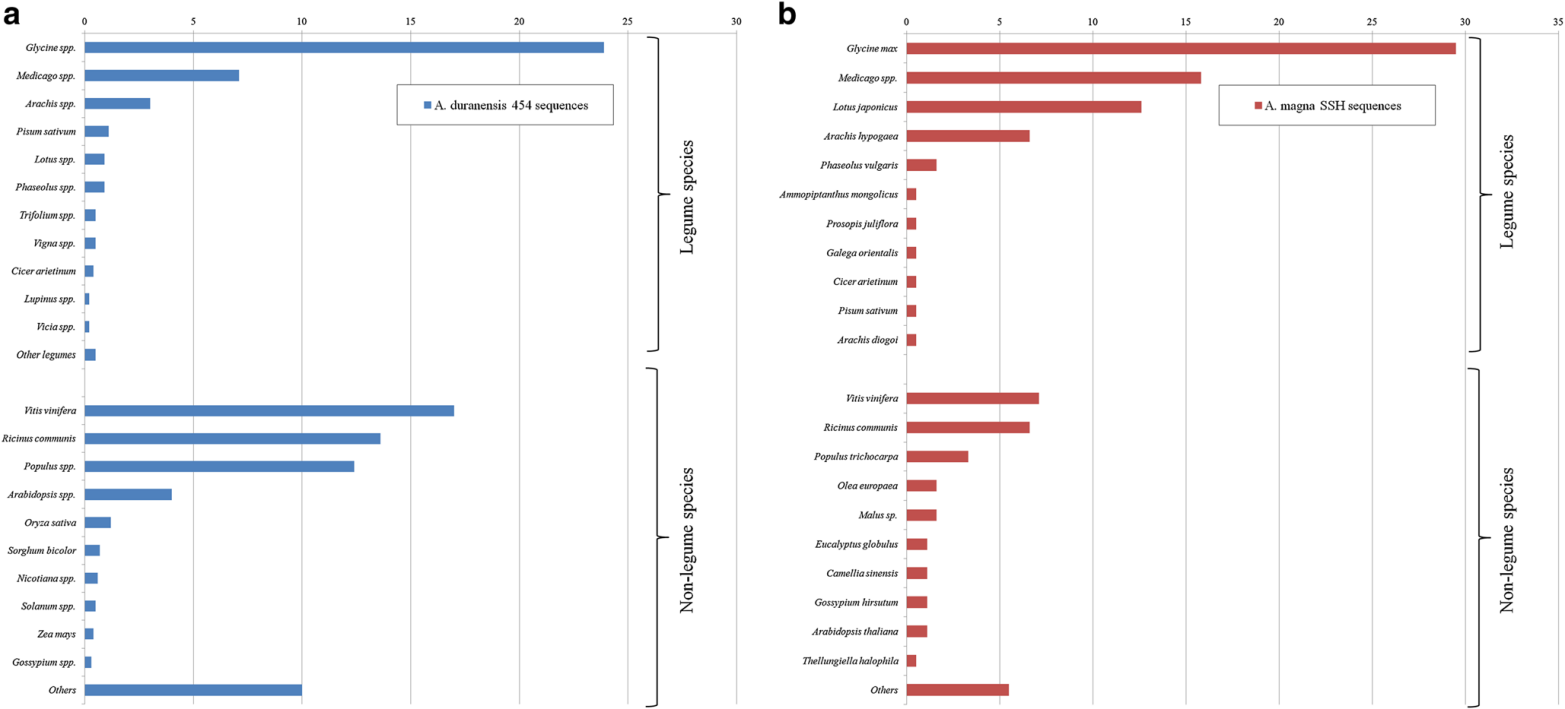
**a**, **b** Distribution by plant species of 12,792 *A. duranensis* contigs (**a**) and 757 *A. magna* unigenes (**b**), according to BLAST hits. Similarities to plant species are determined and affected by the data available in the relevant public databases. *Bar values* correspond to the percentage of unigenes in relation to the total number of annotated sequences of *A. duranensis* 454 and *A. magna* SSH sequences

Transcriptome 454 sequence data of *A. duranensis* roots under water deficit previously produced by this group (Guimaraes et al. 2012) was further investigated in order to identify genes involved in drought stress responses. From the 12,792 high confident consensus sequences (contigs) generated by de novo assembly, 7483 (58 %) showed significant functional annotation of which 40 % are legume homologous with the highest similarities to *Glycine* (24 %) and *Medicago* (7 %) (Fig. 3). This is probably due to the large number of *Glycine* sequences available in public databases (Li et al. 2012) and this being one of the most closely related genus to *Arachis* among the papilionoid legumes, especially compared to the model legumes *Medicago* and *Lotus* (Bertioli et al. 2011). Within species of the genus *Arachis*, transcript similarity was only 3 % (Fig. 3), reflecting the relatively low amount of sequence data currently available. Nevertheless, the high degree of sequence homology with peanut suggests a close evolutionary relationship between peanut and wild *A. duranensis*, its most probable donor of the maternal genome (Robledo et al. 2009).

From the 12,792 *A. duranensis* generated contigs, 5236 (41 %) were exclusively present in the DS library, 2511 (20 %) only in the WW library, and the remaining 5045 (39 %) in both libraries. Additional in silico analysis estimated gene abundance per library, using the normalized raw number of reads mapped in contigs, according to Beneventi et al. (2013). Normalized read count assignment represents the abundance of each contig in the sample, and it can be suitable for estimation of transcript levels in organisms without reference genome, such as *Arachis*, when gene models are not known (Jain 2012)*.* In the present analysis, only contigs with over ten reads (11.8 %) after normalization and with a fold change >2.0 or <−2.0 were taken into account and ranked according to the number of reads in each library. Based on this, 677 contigs were considered positively regulated under stress condition, as they showed a higher number of reads in the stressed library (26 exclusive) than in the control, while 837 contigs (30 exclusive) were negatively regulated, as they were more abundant on the control library. Thirty-one *A. duranensis* candidate genes with top ranked differentially expressed contigs and which also had a functional annotation related to drought-tolerance were selected for further expression analysis by qRT-PCR. Among them, 18 were associated with drought-responsive genes and 13 to transcription factors (TFs) involved in abiotic stress signaling (Table 1).

**Table 1:** *A. duranensis* 31 selected contig descriptions, putative in silico expression pattern (fold change) during gradual drought stress, and predicted proteins (BLASTX)

Gene abbreviation	NCBI ID	No. of reads/contig	Putative fold change^a^	Description from nr hit (BLASTX)	Accession ID	Plant species
Ad*P450*	JR333734.1	200	165.22	Cytochrome P450	XP_003537622.1	*Glycine max*
Ad*EXLB*	JR342047.1	162	121.34	Expansin-like protein	XP_003611388.1	*Medicago truncatula*
Ad*NIT*	JR334709.1	387	−112.56	Nitrilase-associated protein	ACF74272.1	*Arachis hypogaea*
Ad*AGP*	JR333972.1	200	−87.41	Glucose-1-phosphate adenylyltransferase small subunit 2	XP_003519654.1	*Glycine max*
Ad*HMBPP*	JR342513.1	64	55.30	4-Hydroxy-3-methylbut-2-enyl diphosphate reductase	XP_003541082.1	*Glycine max*
Ad*VF*	JR334667.1	116	50.61	vf14-3-3d protein	BAB17822.1	*Vicia faba*
Ad*CICLE*	JR341656.1	52	41.23	Hypothetical protein CICLE	XP_006443352.1	*Citrus clementina*
Ad*CGI*	JR344008.1	58	−40.11	CGI-like protein	KEH28528.1	*Medicago truncatula*
Ad*PG*	JR344000.1	16	2.38 (exclusive)	Prostaglandin E synthase 2	KHN45935.1	*Glycine soja*
Ad*ALDH*	JR334024.1	77	−6.25 (exclusive)	Aldehyde dehydrogenase	XP_003528912.1	*Glycine max*
Ad*APR*	JR334628.1	290	4.16	5′-Adenylylsulfate reductase 3, chloroplastic-like	XP_008224688.1	*Prunus mume*
Ad*PTS*	JR342919.1	20	2.38	Proteasome subunit beta	XP_003532008.1	*Glycine max*
Ad*STPP*	JR344128.1	50	2.62	Serine/threonine-protein phosphatase	XP_003536224.1	*Glycine max*
Ad*ARF*	JR343401.1	15	3.30 (exclusive)	ADP-ribosylation factor protein	XP_003607403.1	*Medicago truncatula*
Ad*NPL*	JR336228.1	38	3.30 (exclusive)	Nuclear protein localization protein	XP_003554491.1	*Glycine max*
Ad*DRPol*	JR333796.1	191	2.79	DNA-directed RNA polymerase II	XP_004493418.1	*Cicer arietinum*
Ad*LLP*	JR343442.1	202	2.77	Legume lectin-like protein	BAN37442.1	*Apios americana*
Ad*RD22*	JR334285.1	849	−2.15	Dehydration-responsive protein RD22	XP_002284439.1	*Vitis vinifera*
Ad*HD*-*ZIP*	JR337754.1	38	3.10 (exclusive)	Homeobox-leucine zipper protein ATHB 6-like	XP_003524258.2	*Glycine max*
Ad*ERF*	JR333375.1	33	2.45	Transcription factor EREBP	CAD56217.1	*Cicer arietinum*
Ad*ARF*	JR339459.1	123	2.35	Auxin response factor	XP_003544394.2	*Glycine max*
Ad*MYC*	JR334826.1	205	−6.52	Transcription factor AtMYC2	XP_002519814.1	*Ricinus communis*
Ad*bZIP1*	JR341217.1	14	−10.13	Basic leucine zipper transcription factor	XP_007042281.1	*Theobroma cacao*
Ad*bZIP*2	JR336137.1	61	−4.90 (exclusive)	Basic leucine zipper transcription factor	XP_007041827.1	*Theobroma cacao*
Ad*bZIP*3	JR343432.1	24	4.25	Transcription factor bZIP109	XP_006572907.1	*Glycine max*
Ad*bZIP*4	JR340355.1	96	10.86	Transcription factor bZIP41	NP_001237222.1	*Glycine max*
Ad*CCAAT*	JR342432.1	66	−10.25	Transcription factor CCAAT	BAG50055.1	*Lotus japonicus*
Ad*HSF*	JR338126.1	82	22.67	Heat shock factor	KHN42077.1	*Glycine max*
Ad*IWS1*	JR334827.1	247	6.69	Interact with Spt6 (IWS1) homolog	XP_003534295.1	*Glycine max*
Ad*MYB*	JR341544.1	65	5.70 (exclusive)	MYB-related protein 30	AHB59616.1	*Arachis hypogaea*
Ad*NAC*	JR341059.1	38	3.30 (exclusive)	NAC-domain-containing protein	NP_001240958.1	*Glycine max*

^a^Fold change = normalized read counts between stressed and control samples. Positive and negative values indicate positive (up) and negative (down) regulation, respectively. (Exclusive) indicates that all corresponding reads derived from a same library, positive values from DS library and negative values from WW library

### *A. magna* Sequence Analysis and Functional Annotation

In silico analysis of the two *A. magna* cDNA libraries revealed 757 high-quality ESTs (Table 2), with an average size of 331 bp, which are available in GenBank dbEST (JZ390113 to JZ390862). *A. magna* ESTs were clustered into 284 unigenes (105 contigs and 179 singletons), with the number of reads per contig ranging from 2 to 56 and a novelty index of 32.8 % (Table 2). Among these unigenes, 194 (68.3 %) had significant BLASTN hits (using default parameters) with at least one contig present in *A. duranensis* 454 libraries, suggesting that *A. duranensis* and *A. magna* share some of the genes involved in the signaling pathways in response to water deficit. BLASTX and Blast2GO algorithms revealed that approximately half of these sequences (48 %) had significant matches to genes encoding proteins with known functions, in accordance to previous *Arachis* transcriptome studies on peanut or AA genome *Arachis* species (Ding et al. 2014; Govind et al. 2009; Guimaraes et al. 2012; Guo et al. 2008; Li et al. 2014; Proite et al. 2007; Ranganayakulu et al. 2011; Tirumalaraju et al. 2011). Therefore, these *A. magna* unigenes represent the first functionally annotated transcriptome analysis for a BB genome of an *Arachis* species.

**Table 2:** In silico analysis of the suppression subtractive hybridization (SSH) sequences from *A. magna* libraries

SSH libraries	Number of ESTs	Number of unigenes	Singletons	Contigs	Novelty index (%)
Stressed library subtracted from control library (STR)	594	246	159	87	38.96
Control library subtracted from stressed library (CTR)	163	38	20	18	21.96
Stressed and control libraries	757	284	179	105	32.80

**Fig. 4:**
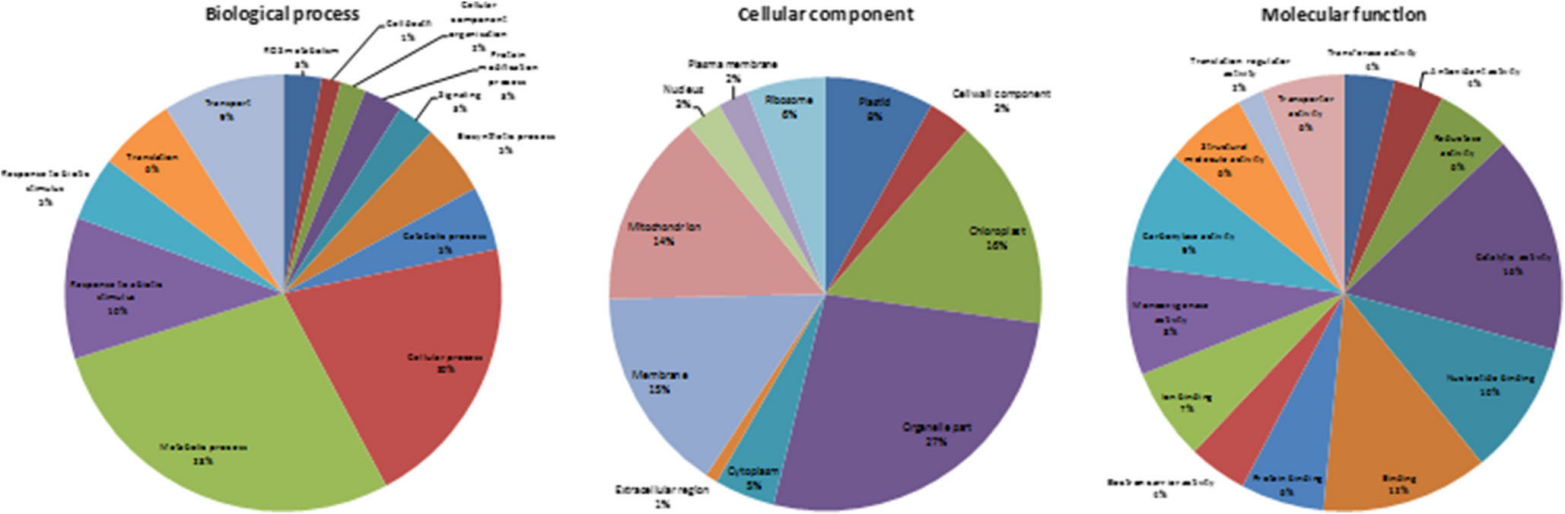
Gene Ontology (GO) classification of the predicted *A. magna* unigenes according to biological process, cellular component, and molecular function using Blast2GO

The identified genes with known function could be then assigned to three broad functional categories, i.e., biological process, cellular component, and molecular function (Fig. 4). The biological process category “metabolic process” was the most prevalent (28 % of sequences), followed by “cellular process” (20 %) and “response to abiotic stimulus” (10 %). In the cellular component category, genes assigned as “organelle part” accounted for the largest group (27 %), followed by “chloroplast” (16 %), “membrane” (15 %), and “mitochondrion” (14 %). For molecular function, the most abundant sequences were associated with “catalytic activity” (16 %), “binding” (12 %), followed by “nucleotide binding” (10 %). Based on GO annotation, *A. magna* ESTs associated to the response to water deprivation and to signal transduction pathways under drought, such as lipocalin, LEA, and dehydrin, could also be identified.

The majority of the *A. magna* annotated sequences (70 %), as for *A. duranensis*, had the best BLASTX hits with legume transcripts, mostly with *Glycine* (30 %), *Medicago* (16 %), *Lotus* (13 %), and *Arachis* (7.1 %) (Fig. 3). Interestingly, subtracted expressed genes of *A. magna* also showed similarity to other legumes, such as *Ammopiptanthus mongolicus* and *Prosopis juliflora*, and to the non-legume *Thellungiella halophila* (Fig. 3), which are emerging as model species for drought and salt tolerance studies (Sawal et al. 2004; Volkov and Amtmann 2006; Zhou et al. 2012).

Among the 105 *A. magna* contigs identified, 87 were predicted to be upregulated (exclusively identified on forward STR library), while 18 were downregulated (reverse CTR library; Table 2), indicating that, under these conditions, drought stress triggered an overall increase in gene expression. No common contigs were shared in forward and reverse libraries, confirming the accuracy of the suppression effect during library construction. This reinforces the relevance of SSH-based transcriptome analysis, even in the current NGS era, as a reliable and efficient approach to enrich and identify specific transcripts for given experimental contrasting conditions, in particular for orphan species such as *A. magna*, with no available transcriptome data. Contigs from both libraries were then ranked according to the number of contributing ESTs (reads per contig), and the 13 most expressed ones, including one unknown protein and 12 showing BLASTX identity with known abiotic-stress-responsive genes, were selected for validation by qRT-PCR (Table 3).

**Table 3:** *A. magna* 13 selected unigene descriptions, putative in silico expression pattern during gradual drought stress, and predicted proteins (BLASTX)

Gene abbreviation	NCBI ID	No. of ESTs/contig	Putative expression pattern^a^	Description from nr hit (BLASTX)	Accession ID	Plant species
Am*DRRP*	JZ390835.1	37	Upregulated	Disease resistance response protein	ABC46711.1	*Arachis hypogaea*
Am*WSD1*	JZ390246.1	13	Upregulated	*O*-acyltransferase WSD1-like protein	KEH30680.1	*Medicago truncatula*
Am*DiP*	JZ390249.1	9	Upregulated	Drought inducible protein	ABW06773.1	*Zea mays*
Am*CA*	JZ390303.1	7	Upregulated	Carbonic anhydrase	XP_006603811.1	*Glycine max*
Am*CAB*	JZ390526.1	6	Upregulated	Chlorophyll a-b binding protein	XP_003536189.1	*Glycine max*
Am*GO*	JZ390375.1	5	Upregulated	Peroxisomal glycolate oxidase	KEH38401.1	*Medicago truncatula*
Am*CAX*	JZ390585.1	4	Downregulated	Ca2+/H+ exchanger	BAA25753.1	*Vigna radiata*
Am*AMT*	JZ390202.1	4	Upregulated	Aminomethyltransferase	XP_006600302.1	*Glycine max*
Am*AiP*	JZ390472.1	4	Upregulated	Aluminum-induced protein	AAQ74889.1	*Gossypium hirsutum*
Am*MET*	JZ390463.1	4	Upregulated	Metallothionein-like protein	AAO92264.1	*Arachis hypogaea*
Am*CDSP*	JZ390484.1	4	Upregulated	Chloroplastic thioredoxin-like protein CDSP32	XP_009106259.1	*Brassica rapa*
Am*TyPP*	JZ390407.1	4	Upregulated	Thylakoid membrane phosphoprotein	XP_002511952.1	*Ricinus communis*
Am*UKN*	JZ390486.1	4	Downregulated	No significant similarity (unknown protein)		

*SSH* suppressive subtractive hybridization

^a^In silico putative expression pattern according to the origin of the sequences: Upregulated sequences are from STR forward SSH library and downregulated from CTR reverse SSH library

### Selection of Candidate Genes by qRT-PCR Analyses

All 31 *A. duranensis* and 13 *A. magna* candidate genes analyzed by qRT-PCR showed specificity of transcript amplification, with high amplification efficiencies (Supplementary Table 1). Actin and ubiquintin previously identified by our group as the most stably expressed genes in *A. duranensis* and *A. magna* subjected to abiotic stress (Morgante et al. 2011) were used as reference genes. Their stable expression levels, regardless of tissue (root and leaf), treatment (control and stress), or collecting point, corroborate their herein usefulness, as well as the need to use at least two reference genes for qPCR data normalization.

**Fig. 5:**
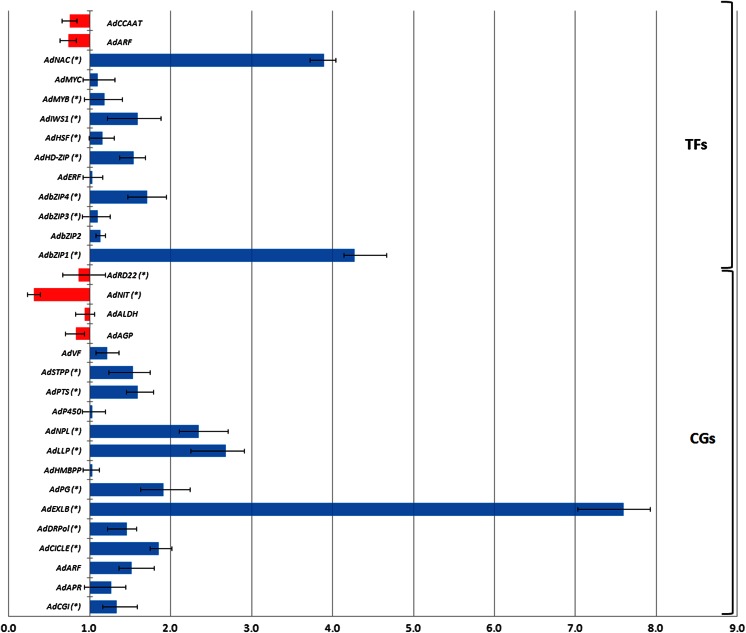
Expression analysis of 13 transcription factors (TFs) and 18 other candidate genes (CGs) by qRT-PCR in *A. duranensis* plants submitted to a gradual water deficit. Axis *X* represents the relative quantification (RQ) between the DS and WW gathered samples, and the *error bars* indicate standard deviation of three replicates. Significantly (*p* < 0.05) upregulated or downregulated genes are indicated by *asterisks*

**Fig. 6:**
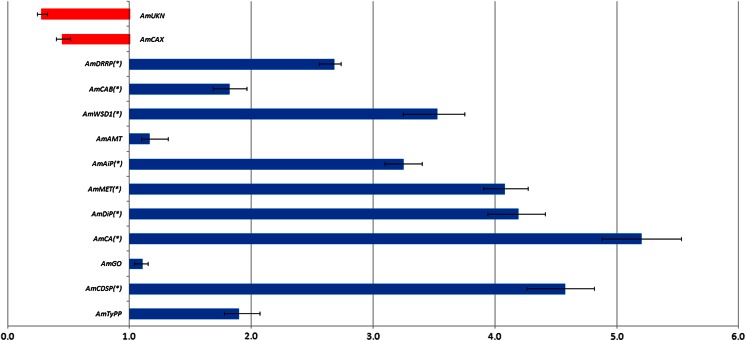
Expression analysis of 13 candidate genes by qRT-PCR in *A. magna* plants submitted to a gradual water deficit. Axis *X* represents the relative quantification (RQ) between the DS and WW gathered samples, and the *error bars* indicate standard deviation of three replicates. Significantly (*p* < 0.05) upregulated or downregulated genes are indicated by *asterisks*

Using pooled samples, the qRT-PCR expression pattern of the 31 *A. duranensis* candidate genes was consistent with in silico analysis, although the latter showed less pronounced changes (Fig. 5; Table 1). These results confirmed the validity of estimating gene abundance using the gene count strategy (Beneventi et al. 2013) but reinforced the need for further validation through accurate in vitro analyses. Likewise, *A. magna* qRT-PCR expression profile data corroborated the subtracted cDNA libraries in silico analysis (Fig. 6; Table 3), as the 11 genes selected from STR library showed a predicted positive regulation, whereas the two genes derived from CTR library were downregulated.

The 13 selected TF genes are representatives of the most ubiquitous TF protein families in *A.* duranensis 454 transcripts (Guimaraes et al. 2012) and are also referred to as potential candidates for drought adaptation improvement (Hu and Xiong 2014), such as *bZIP*, *MYB*, *ERF*, *bHLH*, *NAC*, and *HSF*. From those, eight showed significant upregulation under the dry-down according to qRT-PCR analysis (Fig. 5) with *bZIP1* and *NAC* displaying the highest levels of expression (4.3- and 3.8-fold increase, respectively). *bZIP* and *NAC* families are highly represented in our 454 *A. duranensis* dataset as 13 and 7 %, respectively (Guimaraes et al. 2012), and also among the ten most represented TF families currently described in the peanut transcriptome (Jin et al. 2014). Under this perspective, *A. duranensis bZIP1* and *NAC* candidates (Ad*bZIP1* and Ad*NAC*, respectively) were selected for a more comprehensive qRT-PCR analysis.

Besides TF genes, another 18 *A. duranensis* candidate genes were also analyzed by qRT-PCR and 11 of them displayed a significant change in mRNA levels under drought imposition (Fig. 5). A gene coding for putative *A. duranensis* Expansin-like B (Ad*EXLB*) displayed the highest (7.6-fold) levels of positive differential expression in DS plants compared to WW, whereas Nitrilase (Ad*NIT*) which decreased nearly 2.3-fold was the only candidate gene that showed a significant downregulation (Fig. 5). These genes are associated with water loss control and growth delay in response to drought perception (Ma et al. 2013; Piotrowski 2008) and were selected for further qRT-PCR evaluation.

Analysis by qRT-PCR of 13 candidate genes from *A. magna* showed that 11 of them displayed an upregulated profile in response to water deficit imposition while the remaining two were downregulated (Fig. 6). However, as also observed for *A. duranensis*, only a few candidate genes (*DiP*, *CDSP*, *MET*, and *CA*) had a high (fold change ≥4) and significant differential level of transcription. As their significant upregulation could be associated with drought tolerance mechanisms, these genes were also selected for qRT-PCR analysis.

### Detailed Expression Profile of Candidate Genes

Additional qRT-PCR analysis was conducted to allow a detailed expression profiling for *A. duranensis* (Adb*ZIP1*, Ad*NAC*, Ad*EXLB*, and Ad*NIT*) and *A. magna* (Am*DiP*, Am*CDSP*, Am*MET*, and Am*CA*) candidate genes. For this, root and leaf samples from *A. duranensis* and *A. magna* at each collecting point were analyzed and expression profiles evaluated along the gradual drought imposition.

The four primers designed for *A. duranensis* candidate genes showed high amplification efficiency (0.87 to 0.98) and a specific peak during melting curve analysis when using *A. magna* cDNA as template. Equally, *A. magna* primers efficiently amplified (0.84 to 0.95) *A. duranensis* samples and resulted in single-peaked melting curves. Moreover, all four *A. duranensis* genes presented at least one contig as orthologous in *A. magna* cDNA libraries and, as expected, the upregulated genes (Ad*bZIP1*, Ad*NAC*, and Ad*EXLB*) in the STR library and the downregulated Ad*NIT* in the CTR library. Similarly, all four *A. magna* genes (Am*DiP*, Am*CDSP*, Am*MET*, and Am*CA*) presented at least one orthologous contig, exclusively in the stressed *A. duranensis* 454 library. These high levels of similarity are expected as previous findings showed an extensive synteny between AA (*A. duranensis)* and BB (*A. magna*) genomes of *Arachis* (Moretzsohn et al. 2009).

**Fig. 7:**
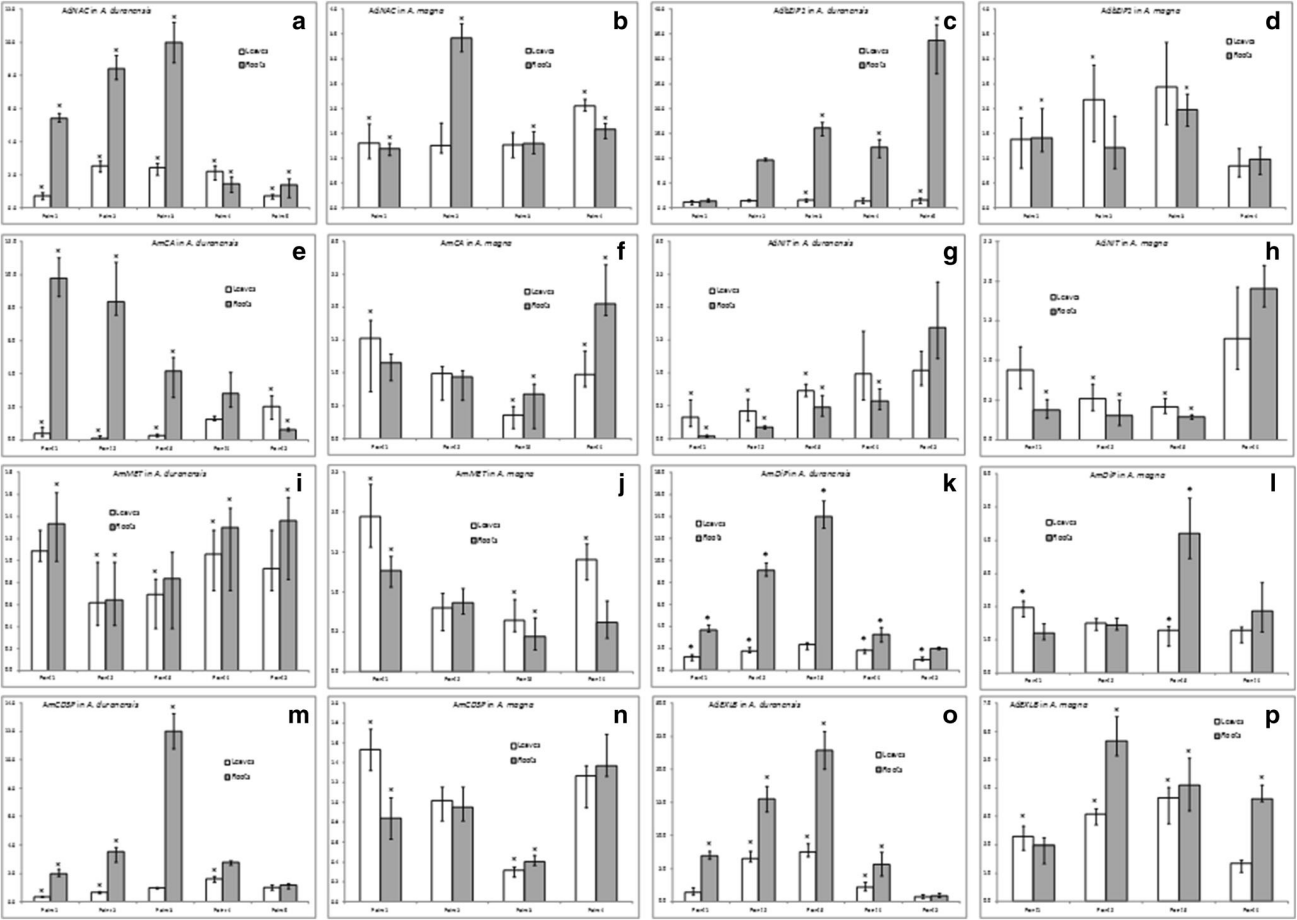
**a**–**p** Expression analysis of eight candidate genes by qRT-PCR in leaves and roots of *A. duranensis* and *A. magna* submitted to a gradual water deficit and rehydration, at different collecting points. Axis *X* represents the relative quantification (RQ) between the DS and WW pools and the mean value of three replicates. Significantly (*p* < 0.05) upregulated or downregulated genes are indicated by *asterisks*

The eight candidate genes exhibited distinctive responses to drought challenge (Fig. 7), and in general, the results corroborated previous in silico and qRT-PCR analyses using pooled samples (collecting points gathered), demonstrating that pools can be a simpler and faster way to select highly expressed candidates among a large number of genes identified using in silico analysis.

Candidate gene expression behavior at different collecting points along the gradual water deficit was, on the whole, similar for both species, as well as during drought recovery steps (Fig. 7). In general, gene expression levels in roots were higher than in leaves, even for the genes selected from *A. magna* SSH leaf libraries. In fact, roots are one of the primary sites of drought perception, which triggers a cascade of gene expression responses, resulting in physiological changes and eventually determining the level of adaptation to the stress in the plant as a whole (Hu and Xiong 2014). Therefore, roots were revealed to be a good target for the study of *A. duranensis* and *A. magna* genes involved in drought perception and key processes underlying plant adaptation and tolerance to water-limited conditions. Proteins coded by the eight candidates genes are involved in these processes by controlling functions related to gene expression regulation, primary metabolism and flux, hormone homeostasis, prevention of cell damage, and protection/adaptation of cell structures and might function to enhance drought adaptation and tolerance in *A. duranensis* and *A. magna*.

#### Genes Involved in Signal Transduction

Ad*NAC* exhibited a significant and gradual enhancement in transcript levels in *A. duranensis* leaves and roots, up to rehydration point 4, when its expression dropped to near the basal level (Fig. 7a). Particularly in roots, this gene reached higher levels of upregulation (10-fold) at the threshold collecting point of the water deficit treatment (point 3 = NTR 0.28; Fig. 7a), when evident root desiccation was observed (Fig. 2b). Conversely, a discreet increase in Ad*NAC* transcripts was observed in *A. magna*, with levels close to 1.2-fold during stress imposition, excepted at point 2, when expression in roots showed a significant upregulation of 3.41-fold (Fig. 7b). After rehydration, Ad*NAC* expression slightly increased in *A. magna* roots and leaves (1.59- and 2.05-fold, respectively). *NAC* is a multifunctional plant-specific TF family, with more than 8000 putative members (Jin et al. 2014), and is involved in regulation of various stress signaling responses, conferring tolerance to abiotic stress in transgenic plants (Puranik et al. 2012). Many studies showed that the overexpression of *NAC*-coding genes could improve drought tolerance and that they are more effective when under the control of stress-responsive or tissue-specific promoters (Hu and Xiong 2014; Nakashima et al. 2014). Moreover, the overexpression of a peanut *NAC* gene in *Arabidopsis* conferred enhanced tolerance to drought and salinity under greenhouse conditions (Liu et al. 2011), corroborating its potential role in signaling stress responses by improving drought tolerance.

Another TF gene analyzed here, Ad*bZIP1*, was also remarkably responsive to the water deficit treatment in *A. duranensis* roots, with its expression rising 16-fold from point 1 to 3 (Fig. 7c) and peaking at 72 h after rehydration, with an upregulation of 33.69-fold. On the other hand, its expression in *A. duranensis* leaves and in *A. magna* roots and leaves seemed to be not, or very weakly, regulated in response to drought or to rehydration, with relative expression ranging from 0.85- to 2.44-fold (Fig. 7c, d). These expression profiles indicated that Ad*bZIP1* is highly involved, at least in *A. duranensis* roots, in the positive modulation of downstream signaling pathways of the drought recovery process and, to a lesser extent, in the response to the water deficit. Members of *bZIP1* TFs, such as AREBs/ABFs, regulate the expression of many genes in the abscisic acid (ABA)-dependent signaling pathway in response to drought and dehydration conditions (Hu and Xiong 2014; Nakashima et al. 2014). Recent studies demonstrated that the expression of an AREB gene isolated from peanut modulated reactive oxygen species (ROS) accumulation and endogenous ABA levels in transgenic *Arabidopsis thaliana*, with improved drought tolerance (Li et al. 2013).

Interestingly, it was demonstrated that members of both TF families here analyzed, *bZIP1* and *NAC*, interact synergistically to control water loss, helping plants to survive under dehydration or osmotic stress conditions (Xu et al. 2013). This observation suggests the cooperative and complex interactions between *bZIP1* and *NAC* regulons. Therefore, the increased expression of Ad*NAC* and Ad*bZIP1* in *A. duranensis* roots in a putative cooperative interaction in response to the imposed gradual water deficit may indicate that these genes are promising candidates for engineering drought tolerance in peanut.

#### Genes Involved in Primary Metabolism

In *A. duranensis*, Am*CA* gene showed an opposite profile in roots compared to leaves (Fig. 7e): when an evident elevation (9.79-fold at collecting point 1) of Am*CA* transcript levels was observed in roots, a remarkable suppression of its levels occurred in leaf tissues (10-fold downregulation at collecting point 2). After re-watering, Am*CA* expression in both tissues returned to the basal levels. However, in *A. magna*, Am*CA* showed a slight upregulation, followed by downregulation in response to drought stress which was recovered after rehydration (Fig. 7f). Carbonic anhydrase (*CA*) is an ubiquitous zinc-containing enzyme which catalyzes the reversible hydration of CO_2_ and has a putative role in the decreasing of photosynthesis under drought conditions (Perez-Martin et al. 2014). Its expression under dehydration and osmotic stress could be species-dependent or even associated with its subcellular localization (Wu et al. 2012). A decrease in *CA* expression during drought stress, followed by an upregulation after rehydration, suggested the involvement of this gene in the regulation of mesophyll conductance to CO_2_ (Perez-Martin et al. 2014), by altering carbon primary metabolism. A negative modulation of Am*CA* expression in both wild *Arachis* along the low water availability is, hence, in accordance with the literature. Nevertheless, the opposite behavior of Am*CA* in *A. duranensis* leaves and roots is a very interesting issue concerning its tissue-specific expression that will be addressed in forthcoming studies.

Ad*NIT* showed the strongest repression levels in response to dry-down imposition in both *A. duranensis* and *A. magna* (Fig. 7g, h). Remarkably in *A. duranensis* roots, transcripts displayed a drastic and significant decrease (25-fold) during initial dry-down stages, as an immediate reaction to drought stress perception (Fig. 7g). As stress progressed, a gradual increase in the expression was observed, achieving a positive regulation (1.69-fold) 72 h after rehydration (point 5; Fig. 7g). In a similar way, the levels of Ad*NIT* transcripts in leaves of *A. duranensis* significantly decreased (3-fold) at the beginning of stress and gradually recovered its basal level 30 min after re-watering (point 4; Fig. 7g). In *A. magna* samples, the repression of Ad*NIT* expression as a possible stress perception seemed to be delayed comparing to *A. duranensis* (Fig. 7g, h), showing a gradual and significant expression decline as the water availability in the soil decreased and reaching the lowest level of expression (2.38- and 3.45-fold for leaves and roots, respectively) at the end of stress (point 3; Fig. 7h). Nitrilase-like proteins (*NIT*) are plant enzymes that hydrolyze organic nitriles to ammonia and corresponding carboxylic acids. NIT is involved in cyanide detoxification and nitrogen recycling and also in biosynthesis of the plant hormone indole-3-acetic acid (IAA), the levels of which could decline in response to drought perception causing growth delay (Piotrowski 2008). The downregulation of Ad*NIT* in response to water deficit stimulus could be associated with the putative role of this enzyme in processes regulating cell proliferation and differentiation in order to maintain a balance with plant cell death (PCD) and also in cyanide detoxification and nitrogen primary metabolism (Doskocilova et al. 2013; Piotrowski 2008). Curiously, NIT interacts with ERF and NAC proteins, which are TF family members involved in the regulation of ethylene and IAA responses as well as in drought stress tolerance (Huh et al. 2012; Xu et al. 1998), as discussed above. The overexpression of *Arabidopsis NIT1* resulted in the suppression of cell growth and increased levels of PCD and, conversely, plants with reduced *NIT1-3* expression of showed diminution of leaf and root growth (Doskocilova et al. 2013). Those results indicate that the inhibition of *NIT* expression trigged by water-limited conditions could help plants in controlling cell growth and root elongation to circumvent drought adverse effects. However, the specific role of NIT in drought responses, involving plant developmental processes, such as cytokinesis and PCD and IAA and ethylene homeostasis, remains to be demonstrated.

#### Genes Involved in Cell Protection and Adaptive Mechanisms

Am*MET* exhibited overall similar responses to drought in leaves and roots for both *A. duranensis* and *A. magna* (Fig. 7i, j). In these species, Am*MET* apparently responds at the beginning of the stress with a modest increase in its expression that changed to a slight downregulation along the treatment followed by the increasing expression to basal levels after rehydration (Fig. 7i, j). Metallothioneins (MET) are metal-binding cytosolic proteins widely distributed in living organisms and implicated in ROS scavenging and metal detoxification homeostasis to protect cells against a variety of stresses (Leszczyszyn et al. 2013). Ad*MET* codes for a putative MET type 3 that is thought to be positively regulated in response to water-limited conditions and thereby plays a role in drought tolerance (Xue et al. 2009), probably by protecting cells and reducing ROS damage during defense signaling. Therefore, the herein Ad*MET* positive regulation, previously indicated by in silico and qRT-PCR analyses conducted with *A. magna* gathered samples (Table 3 and Fig. 6, respectively), occurred indeed only at the beginning of the stress (point 1) in both *A. duranensis* and *A. magna* samples, as demonstrated in the point-by-point qRT-PCR analysis (Fig. 7i, j).

The other *A. magna* candidate gene, Am*DiP*, displayed a marked increase in transcript level in *A. duranensis* roots, with the peak (14.01-fold) at the threshold collecting point of the treatment, followed by a decrease to basal levels 72 h after rehydration (Fig. 7k). Am*DiP* presented a proportionate expression pattern in *A. duranensis* leaves, but with lower levels than in roots (Fig. 7k). For *A. magna*, the upregulation peak of Am*DiP* (4.20-fold) in roots was at the threshold point of the treatment, while in the other root collecting points and in leaves, this gene expression remained close to the basal level (1.21- to 1.98-fold) (Fig. 7l). Drought-induced proteins (DiP) are small proteins present in several plant species, whose expression is highly modulated by abiotic stress (Kalifa et al. 2004). Am*DiP* has high sequence similarity with a group of DiP plant proteins that have the same expression profile as the one herein determined for *Arachis* spp., i.e., highly activated under water-limited conditions. DiPs are regulated by an ABA-dependent pathway whenever the plant is in a stressful environment, and it is associated with chromatin protection or signal transduction (Kalifa et al. 2004), but its biological function remains unclear.

Am*CDSP* exhibited a very similar expression profile to Am*DiP* in *A. duranensis* roots, with an intense increase in transcript levels in response to drought imposition (peak of 12.02-fold at threshold point) and basal expression return during rehydration (Fig. 7m). Conversely, expression of Am*CDSP* in *A. duranensis* leaves displayed a significant decline (2.94-fold) at the drought beginning, followed by a gradual recovery to basal levels just after re-watering which was maintained (Fig. 7m). In *A. magna*, Am*CDSP* showed a different expression behavior to *A. duranensis*, with a gradual expression decline during drought imposition (3.14- and 2.47-fold, for leaves and roots, respectively) at the threshold stress collecting point (Fig. 7n). After 5 h of rehydration, Am*CDSP* expression recovered to the basal levels in both tissues. *CDSP* gene codes for Chloroplast Drought-induced Stress Protein described as highly responsive to drought, but with no attributed function (Broin et al. 2000). AmC*DSP* showed a high degree of similarity to the *A. thaliana* thioredoxin-like protein CDSP32 which is supposed to participate in the prevention of cell damage and protection of chloroplast structures upon oxidative stress within the chloroplast during water deficit (Broin et al. 2000). This protective function could be extended to Am*CDSP* since its expression pattern observed in *A. duranensis* roots is in accordance with CDSP32 previously described behavior under drought stress.

Notably, among these eight selected candidates, Ad*EXLB* showed the highest transcriptional induction in response to the progressive water deficit in soil (Fig. 7). Although at different magnitude, its expression profile in *A. duranensis* roots and leaves was very similar to *A. magna*, with a strong induction in response to drought stress followed by significant decrease after rehydration (Fig. 7o, p). In *A. duranensis*, Ad*EXLB* expression reached the maximum level of induction (22.87- and 7.54-fold in roots and leaves, respectively) at the threshold collecting point of the stress treatment (point 3; Fig. 7o). Upon 72 h of rehydration, its transcriptional levels in DS plants decreased drastically, recovering to its basal level, i.e., similar to WW plants (Fig. 7o). In *A. magna*, Ad*EXLB* expression showed the same behavior, but its expression peak occurred at point 2 in roots and at point 3 in leaves (5.68-fold and 3.65-fold, respectively; Fig. 7p). After re-watering, expression in leaves decreased to basal levels, whereas in roots, it maintained a similar expression (3.63-fold) to the threshold collecting point of the stress treatment. This suggests that roots need more than 5 h of rehydration to recover Ad*EXLB* basal expression. *EXLB* gene is a member of the Expansin superfamily of plant cell wall-loosening proteins involved in many developmental processes as well as in adaptive responses to mechanical or environmental stimuli. The involvement of genes from the Expansin superfamily in cell wall plasticity is associated with the control of water loss, probably promoting cell wall relaxation aiming cell turgor adjustment, as shown in transgenic drought-tolerant plants obtained by overexpressing Expansin-coding genes (Li et al. 2011; Lü et al. 2013; Ma et al. 2013). Studies conducted in many plants, including the resurrection plant *Craterostigma plantagineum*, also showed increasing expression of Expansins at early stages of dehydration (Choi et al. 2006). Recent genome-wide characterization of Expansin genes in plants revealed that the Expansin like-B (*EXLB*) gene here identified is not well characterized and, together with Expansin like-A, one of the less represented member of the Expansin superfamily (Zhu et al. 2014). Since EXLB amino acid sequences are very divergent in plants, it was suggested that its action mode could differ from that of other superfamily members. Moreover, our preliminary studies indicated that the increase in Ad*EXLB* expression could be associated with more conservative transpiration behavior in nine wild *Arachis* species submitted to water-limited conditions, when compared with cultivated genotypes (not published). Therefore, EXLB became a potential target for the enhancement of drought tolerance in transgenic plants through cell dehydration control via turgor upholding and further analysis is underway.

## Conclusions

Drought stress in plants is a complex issue and often involves an intricate chain orchestrated by physiological events that affect transcriptional and translational regulation in plants. Different plant genotypes often harbor diverse mechanisms of response and adaptation to drought, which might include the expression of a distinct set of genes that trigger changes in specific biochemical pathways and structural arrangements. Wild *Arachis* species (*A. duranensis* and *A. magna*) potentially harbor improved performance under abiotic stress since their native areas are water-limited environments, making them interesting genotypes for drought-related gene discovery. In order to better address the multiplicity of biological processes involved in drought perception and responses, the transcriptome profile of these two wild species was analyzed at different stages of a gradual water deficit, followed by rehydration. Regardless of the differences in the methodological conception in the construction and sequencing of the libraries and differences between AA (*A. duranensis*) and BB (*A. magna*) genomes, these two species have a high number of common genes identified as differentially expressed, suggesting a similar transcriptional modulation in response to water deficit. Moreover, from those, eight candidate genes shared similar expression profiles in response to drought challenge and recovery. This included genes involved in signal transduction, which were highly induced by drought perception or after rehydration in *A. duranensi*s roots, such as *NAC* and *bZIP1*. Other genes that better enable plants to survive under stress conditions, including those involved in the adaptation of primary metabolism, such as *CA* or *NIT*, or coding for proteins implicated in cell protection or adaptation mechanisms, like *CDSP*, *DiP*, or *EXLB*, were also identified in both *A. duranensis* and *A. magna*. This enabled the unraveling of novel promising candidates that might enhance drought tolerance by gene manipulation in bio-engineered crops, particularly peanut and other legumes.

Despite recent efforts of the peanut scientific community, the extension of *Arachis* genomic resources is still limited, hindering *Arachis* gene expression dynamics characterization. Thus, besides a valuable resource for gene discovery, this transcriptome survey of wild *Arachis* species also contributes to a better understanding of gene modulation in response to water deficit and rehydration, providing new tools for peanut breeding programs, such as the characterization of new alleles and development of molecular markers associated with drought stress.

## Electronic supplementary material

Supplementary Table 1 (DOCX 19 kb)
