# Supplementary material for: Transcriptome Profiling of Wild Arachis from Water-Limited Environments Uncovers Drought Tolerance Candidate Genes
Source: Plant Mol Biol Report. 2015 Apr 11;33:1876–92. doi: 10.1007/s11105-015-0882-x (PMC4695501; doi:10.1007/s11105-015-0882-x)
Supplement: Supplementary file 1 — (DOCX 19 kb) [file 11105_2015_882_MOESM1_ESM.docx]

**Supplementary Table 1:** Genes and primers used for qRT-PCR analysis.

| **Gene Abbreviation** | ***Arachis* species** | **Primer sequence Forward/Reverse** | **Amplicon size** | **PCR efficiency** |
| --- | --- | --- | --- | --- |
| *ACT1* | *A. magna* | TGGTCTCGGTTTCCTGAGTT/AATACCACTCCAAAGCAAACG | 114 | 0.991 |
| *ACT2* | *A. hypogaea* | GAGCTGAAAGATTCCGATGC/GCAATGCCTGGGAACATAGT | 178 | 0.990 |
| *UBI2*^a^ | *A. hypogaea* | AAGCCGAAGAAGATCAAGCAC/GGTTAGCCATGAAGGTTCCAG | 145 | 0.992 |
| Ad*P450* | *A. duranensis* | ACTCTGCGTTGATCATGACG/CCAACTTCGAGAAGCTCACC | 154 | 0.992 |
| Ad*EXLB* | *A. duranensis* | TGGGTTCTCAACATCAAACACT/CGCAGCTACAACCCACACTA | 149 | 0.974 |
| Ad*NIT* | *A. duranensis* | ACCTCTTTGGTGGTCCTCCT/GCCACTCCAACATTGTCAAG | 187 | 0.993 |
| Ad*AGP* | *A. duranensis* | ACGCTCCCTCTCATTCTCCT/GCATCGGGATCAAGACAAGT | 158 | 0.982 |
| Ad*HMBPP* | *A. duranensis* | ATTCTTTGCTCGCTGTCGAT/CACGATTTGTGATGCCACTC | 173 | 0.979 |
| Ad*VF* | *A. duranensis* | GGCAAAGAAACACCAAAAGC/AGGGACAACCTCACCTTGTG | 165 | 0.992 |
| Ad*CICLE* | *A. duranensis* | ACAGCACAAGGGTGAAGGAT/AGGTCCTTGCTCTCAGTCCA | 168 | 0.992 |
| Ad*CGI* | *A. duranensis* | TCAAGGGAAAGGCACTTGAT/TCGCATTTTGATCATCCGTA | 159 | 0.988 |
| Ad*PG* | *A. duranensis* | TACAGTGCTGCTCGCTCATC/ACTGTTTCCGAGGCTCTTGA | 164 | 0.963 |
| Ad*ALDH* | *A. duranensis* | AAAAGGCCTTCATATAGCTCCA/ATGAGTGAAGGGTTAGGTGTGG | 94 | 0.987 |
| Ad*APR* | *A. duranensis* | GGCCATGAGAACATTCGTTT/GAATGCCCTCAGGTGATGAT | 162 | 0.997 |
| Ad*PTS* | *A. duranensis* | TGGGTGGTCTTGACAATGAA/CAGCCAATGGAGTAACAGCA | 185 | 0.979 |
| Ad*STPP* | *A. duranensis* | GCCTCTAGAGATTCTTCCCACA/AGGCTTTCTATGGAAGAAGACG | 73 | 0.939 |
| Ad*ARF* | *A. duranensis* | TTTGTTGGCAAACACCAAGA/GGATGTCGGAGGTCAAGAGA | 185 | 0.913 |
| Ad*NPL* | *A. duranensis* | ATCAAGGACGACGATGATCC/CGGTTCTCAATGGGAAATGT | 157 | 0.976 |
| Ad*DRPol* | *A. duranensis* | AAGCGATGATGGTGGAGAAC/TTCAGAAGCAGGGATGGAGT | 160 | 0.940 |
| Ad*LLP* | *A. duranensis* | TTGAGCTGAAAAGCGAAGGT/AGGCAGTGCCAAATTCTCAG | 173 | 0.963 |
| Ad*RD22* | *A. duranensis* | TTGGGTGCCCGGATCTTACT/TTGATCCCCATACATCAAACC | 169 | 0.948 |
| Ad*HD-ZIP* | *A. duranensis* | CATGTCTTCGTGTGGTGGTG/AGGGTTGGTGCTTGTTCATC | 197 | 0.991 |
| Ad*ERF* | *A. duranensis* | CGAGGAGCTTGCAGATATGG/TTGGGTTTCCAACATCCTGT | 127 | 0.983 |
| Ad*ARF* | *A. duranensis* | CCCTCAAATTGTTTGAACTCG/GAGCATTCTGCCGCACTTCA | 177 | 0.989 |
| Ad*MYC* | *A. duranensis* | TGGTTCTTCTTGCTGCATTG/TCCTCCTCCTCTTCCAGACA | 140 | 0.962 |
| Ad*bZIP1* | *A. duranensis* | GGCGCTCATCGAGTGATTCT/ACATGGGCATATGACGAACA | 164 | 0.991 |
| Ad*bZIP2* | *A. duranensis* | GAAGGCAATAGACCCGATCA/ATTAGGGGCAGGATTGAAGG | 147 | 0.932 |
| Ad*bZIP3* | *A. duranensis* | GCGTACCTCATCGACTTCGT/TGTTTCCGCAATGTCTTTGA | 147 | 0.888 |
| Ad*bZIP4* | *A. duranensis* | CGGAAAGTCCACTGACATCC/AAGCCATGACGGAGACTGAG | 122 | 0.955 |
| Ad*CCAAT* | *A. duranensis* | AAAAGCCTCAAGCAGCCATA/TTATGCGTCCCGAAATTTGA | 161 | 0.890 |
| Ad*HSF* | *A. duranensis* | ATGAAGCCGGAGGAACCTCT/GGGATGCTTCTCATGTCGAT | 125 | 0.915 |
| Ad*IWS1* | *A. duranensis* | TCCTCTTCACCTTCCTCTGC/TGGTGTTAGGAACGTGGATG | 125 | 0.804 |
| Ad*MYB* | *A. duranensis* | CTTCTTCAGTCCAAGACACA/CTGGGTATGATGGGTTTAAAGG | 100 | 0.939 |
| Ad*NAC* | *A. duranensis* | GAAGCTTCGGAACTGAATCG/CAGTACCGTCGTGAACCAGA | 181 | 0.933 |
| Am*DRRP* | *A. magna* | GTTGCTCAGGAAATGGTGGT/TTGTTCTTGCAGTGGTCGTC | 112 | 0.989 |
| Am*WSD1* | *A. magna* | GACATTGCAGAGGCACGTAA/GGAATTGGCACACCTGAAAC | 151 | 0.986 |
| Am*DiP* | *A. magna* | ATGCTCATGGAAGGCAAAAC/CGAGGCAAAGAAAGATCCAG | 102 | 0.932 |
| Am*CA* | *A. magna* | TCCCACCATATGACCAGACA/GCTCCATCGTATGGGAAAGA | 150 | 0.981 |
| Am*CAB* | *A. magna* | TGAGCAAGTTGTTGCCAAAG/GTTTGCCATGCTTGGTTTCT | 103 | 0.994 |
| Am*GO* | *A. magna* | CTCTGCCTTGGAAGAGGTTG/CTTCAGCAGCCAAGGAGAAC | 157 | 0.998 |
| Am*CAX* | *A. magna* | GGTCCCTTGTGAGATGAAGG/GGTGGATGGATGGATGATAGA | 99 | 0.996 |
| Am*AMT* | *A. magna* | GGCAACAACATCGGAGAAAT/ACTTTGGTTCCTGCCTTGTG | 106 | 0.982 |
| Am*AiP* | *A. magna* | TGTGGAAACCCCTCTTCAAC/CCAGCTGAGGAGGAGGAAAT | 102 | 0.987 |
| Am*MET* | *A. magna* | ACTTGCACTTCCCATCGTTC/GTGCGTGAAGGGAAACAAAT | 109 | 0.999 |
| Am*CDSP* | *A. magna* | GCGACCTCTGCAAAAGAGAG/GAATGGCTGTCCCCGTAGTA | 138 | 0.984 |
| Am*TyPP* | *A. magna* | GGGTCCCAAAAACTTTGTCT/GCATCCCACTTTTCCTTCAA | 104 | 0.994 |
| *AmUKN* | *A. magna* | TGCTACATCCTCCTCAACACC/AGAAACACTGGCTGTAAAAG | 106 | 0.981 |

^a^ ([Luo et al. 2005](#_ENREF_1))

Luo M, Dang P, Bausher MG, Holbrook CC, Lee RD, Lynch RE, Guo BZ (2005) Identification of transcripts involved in resistance responses to leaf spot disease caused by *Cercosporidium personatum* in peanut (*Arachis hypogaea*). Phytopathology 95:381-387
